# The Addresses at the Belfast Medical Meeting

**Published:** 1909-08-07

**Authors:** 


### The Addresses at the Belfast Medical Meeting.

Last week we published a brief appreciation of this year's President of the British Medical Association, who was installed into his office during last week's annual meeting in the city of Belfast. Since those lines were written Sir William Whitla has delivered his Presidential Address before a large and most distinguished audience, and the other principal addresses of the Conference have also been delivered and reported throughout the British Press. Not only the President's Address and the popular lecture, but also the addresses in medicine and surgery, dealt with matters of great medical interest and importance, but also touched upon various matters of medico-social significance which make a direct appeal to the attention of educated laymen and students of social progress. With Sir William Whitla's "Survey of the State of Medical Education," and its strong and reasonable advocacy of reform in the students' curriculum, we propose to deal fully in our forthcoming annual Educational Number which will appear on Saturday, September 4. This will be a suitable opportunity for a careful inquiry into the groundwork of Sir William Whitla's indictment, and for comment and criticism of his proposals for reform in the methods and principles of medical education. The needs of to-day and the possibilities of to-morrow seem incompatible with any fixed and dogmatic outlook upon medical education; and, therefore, when a teacher and physician of high authority and long and wide experience speaks out his mind upon the flaws in the current curriculum, it is clearly necessary to give close attention to his words.

The addresses in medicine and surgery were respectively entrusted to Dr. R. W. Philip, of Edinburgh, and Mr. A. E. Barker, of London. Each chose a topic with which he has long been identified and upon which he is entitled to speak with certainty and conviction. Dr. Philip has long been regarded by Scottish practitioners, and particularly by those in touch with his work in Edinburgh, as the leading authority of that city upon pulmonary tuberculosis, and not least in its clinical aspects. Therefore it was well that he decided to address the Belfast meeting upon the subject of "Progressive Medicine and the Outlook on Tuberculosis," and thus survey one of the greatest and most far-reaching problems of the present and the future. Dr. Philip cast his mind's eye backward over the past 35 years, and pondered over "the extraordinary revolution of thought which has occurred in little more than a generation" upon tuberculosis, its origin, nature, and prevention; and his address crystallised the progress which has been made in his own time in every department of the anti-tuberculosis campaign. Particularly interesting and valuable was his exposition and advocacy of "The Great Principle of Aerotherapy," and we have been pleased to hear this point emphasised in the non-medical press, and we hope that the echoes will not soon die away in the public mind. It is reasonable to foresee in a future and widespread practical acceptance by the public of the curative and preventive value of the open-air principle of treatment at least a partial solution of the tuberculosis problem.

The surgical address also was devoted to a survey of recent progress in thought and technique; and, just as Dr. Philip took Tuberculosis, so Mr. Barker took Intestinal Surgery as a means of illustration. In each case the special field of activity chosen may reasonably be considered something more than typical of the immense general medical and surgical progress of the past 30 or 40 years. Moreover, just as the discovery of the tubercle bacillus and the complete acceptance of aerotherapy have revolutionised the fight against tuberculosis, so the advent of anæsthesia and of the antiseptic method of wound treatment have revolutionised abdominal surgery. Thus there are many points of similarity, both interesting and profitable to discover, between these two addresses; and the British Medical Association no doubt feels much satisfaction in the substantial value of this year's principal orations.

